# Initial Response to Program, Program Participation, and Weight Reduction Among 375 MOVE! Participants, Augusta, Georgia, 2008–2010

**DOI:** 10.5888/pcd13.150598

**Published:** 2016-04-21

**Authors:** Jane T. Garvin, Dale Hardy, Hongyan Xu

**Affiliations:** Author Affiliations: Dale Hardy, Augusta University, Augusta, Georgia; Hongyan Xu, Medical College of Georgia, Augusta University, Augusta, Georgia.

## Abstract

**Introduction:**

Obesity management guidelines specify initial goals for participation and weight reduction for the first 6 months of a weight-reduction intervention, but guidelines do not specify when to assess early response and make adjustments. We aimed to determine whether very early or early weight reduction in the weight-reduction program MOVE! predicted later participation or achievement of weight-reduction goals.

**Methods:**

Using clinical data from 375 MOVE! participants enrolled from July 2008 through May 2010, we examined program participation and weight reduction. Very early response was defined as achieving a weight reduction of 0.5% or more at week 2, and early response was defined as achieving weight reduction of 1.0% or more at week 4. Success, or achievement of weight-reduction goal, at 6 months, 1 year, and 2 years was defined as a weight reduction of 5% or more. Participation was assessed according to the number of sessions attended within the first 6 months of program enrollment; attendance of 14 or more sessions was classified as high-intensity participation.

**Results:**

Very early responders were more than 5 times as likely (odds ratio [OR] = 5.46; 95% confidence interval [CI], 1.69–17.71; *P* = .005) and early responders were more than 10 times as likely (OR = 10.76; 95% CI, 2.64–43.80; *P* = .001) to achieve the 6-month weight-reduction goal as participants who were not very early responders or early responders, respectively. Early responders were almost 7 times as likely to achieve the 1-year weight-reduction goal (OR = 6.96; 95% CI, 1.85–26.13; *P* = .004). Neither very early nor early response predicted participation, high-intensity participation, or success at 2 years.

**Conclusion:**

This research supports the predictive value of very early response and early response to MOVE! on weight-reduction success at 6 months; early response also predicted 1-year success, suggesting that the 2-week point may be an ideal time to assess initial response and make intervention adjustments.

## Introduction

Weight reduction is a challenge for health care providers and patients (1). Both want to know quickly if an intervention is likely to yield successful results. Weight-reduction success in a comprehensive lifestyle intervention is defined initially as achieving at least a 5% reduction from baseline to the end of 6 months (1,2). The Obesity Society guidelines recommend high-intensity treatment, defined as attending at least 14 sessions of a weight-reduction intervention during 6 months, for people with a body mass index (BMI, in kg/m^2^) of 30 or more and for people with a comorbid condition and a BMI of 27 or more, but the guidelines do not specify an earlier time at which to assess participant response and make adjustments to the intervention (1). Waiting 6 months to determine if the lifestyle intervention alone will yield the desired outcome is not ideal, given the epidemic of obesity and its many comorbid conditions, including diabetes, hypertension, and dyslipidemia (1). As a result, researchers are interested in identifying early or very early predictors of success in comprehensive lifestyle intervention programs.

Early success is an important predictor of later weight reduction. Research indicates that early success with weight reduction predicts later success. Several studies examined initial response to weight-reduction interventions as a predictor of later achievement of weight-reduction goals. Initial response in these studies was defined in multiple ways, including various descriptions of weight reduction at various times (3). For lifestyle interventions, definitions included weight reduction at 6 months (4), percentage of weight reduction at 1 month (5), and 2% weight reduction at 1 month (6). When medication was added to the lifestyle intervention, the definition was 5% weight reduction at 12 weeks (7). We used a simple formula — weight reduction of 1.0% or more at the end of 4 weeks (1 month) — to achieve our primary study objective: to determine if an early response would predict successful weight reduction (5% or more) at 6 months among participants in a weight-reduction program. We examined data from the comprehensive lifestyle weight management program MOVE!, which is offered to US veterans. A secondary aim was to determine if achieving a weight reduction of 0.5% or more at 2 weeks, a very early response, would predict successful weight reduction. These simple formulas could be applied to the clinical setting if they predict weight-reduction success.

We hypothesized that participants who were experiencing a weight reduction with the lifestyle intervention would continue to attend sessions while those who were not experiencing weight reduction would not return. Therefore, we also examined if very early response or early response to the MOVE! program predicted the level of participation or high-intensity participation.

## Methods

In this secondary analysis, we used data from the MOVE! program, described in detail elsewhere (8–10). Briefly, in this intervention, all participants received an initial assessment, written materials, and a pedometer. A nurse, dietitian, physical activity specialist, and psychologist offered face-to-face group sessions on diet, physical activity, behavior change, and use of the Veterans Affairs Medical Center’s (VAMC’s) online self-management system for accessing health care resources. Veterans participated in as many or as few of these sessions as they desired.

For this study, we used clinical data from electronic health records of MOVE! program participants enrolled from July 1, 2008, to May 31, 2010, at the Charlie Norwood VAMC in Augusta, Georgia. We examined data only for participants who met the eligibility criteria for enrollment in a high-intensity intervention (1): 1) having a body mass index of 30 or more or 2) having a body mass index of at least 27 and at least 1 of 3 comorbid conditions (diabetes, hypertension, or dyslipidemia). Of 404 MOVE! participants, 27 did not meet the inclusion criteria, 2 were excluded because they died within 2 years of enrollment, and the remaining 375 (93%) met the eligibility criteria and were included. After entering the MOVE! program, participants were followed for 2 years. Participants were community-dwelling veterans (ie, not residing in a hospital or an institutional facility). 

All data were retrospectively extracted from electronic health records and entered directly into a statistical software program. We extracted data on the following baseline demographic variables: age, sex, and race/ethnicity. We extracted data on height, in inches, and weight, in pounds, at enrollment, converted height to meters and weight to kilograms, and calculated baseline BMI according to these measurements to determine eligibility in the study. Data on the existence of a comorbid condition were also collected at baseline. We extracted data on weight at the following additional points after enrollment: 2 weeks, 4 weeks, 6 months, 1 year, and 2 years. Participants were not required to weigh in at these time points, so data were not available on all participants at each point.

Participants who achieved weight-reduction success were defined as responders; weight-reduction success at 6 months, 1 year, and 2 years was defined as weight reduction of 5% or more from baseline. Participants who did not achieve weight-reduction success at any point in the program were defined as nonresponders. Initial response was further categorized as early responders or very early responders. Very early response was defined as weight reduction of 0.5% or more from baseline to the end of week 2. Early response was defined as weight reduction of 1.0% or more from baseline to the end of week 4. 

Data on participation were extracted from the electronic health record and assessed according to the number of group sessions attended. We examined the total number of group sessions attended by participants as well as the number of participants who attended 14 or more sessions within the first 6 months of enrollment. We used this cut point because of the definition of high-intensity participation provided by the 2013 guidelines for overweight and obesity management (1). The study was approved by the VAMC and university institutional review boards.

For statistical analyses, we used SPSS version 22 (IBM Corporation). The likelihood of achieving successful weight reduction was determined by using logistic regression models. Models examined early and very early response. Control variables in the models included age, sex, race, and program participation (ie, number of group sessions attended). Previous reports suggested that these variables predicted weight reduction for MOVE! participants (8,9,11–13). Program participation was examined by using linear regression; high-intensity participation was examined by using logistic regression. Significance was defined as *P* ≤ .05 for all analyses.

## Results

The 375 MOVE! participants ranged in age from 21 to 81 years and had a mean age of 56.4 (standard deviation [SD], 11.2) years. Seventy-seven participants were female (20.5%). More than half (58%; n = 217) were black; 42% (n = 158) were white; no other groups were represented in the sample; less than 2% were Hispanic, 96% were non-Hispanic, and 2.1% were recorded as unknown ethnicity. Body weight ranged from 148.9 to 458.8 pounds with a mean of 240.2 pounds (SD, 43.9 lbs); BMI ranged from 27.0 to 62.7 with a mean of 35.4 (SD, 5.6). At baseline, almost one-third (31.5%; n = 118) had diabetes; almost two-thirds had hypertension (62.7%; n = 235); and more than half (55.2%; n = 207) had dyslipidemia. We found no significant differences in baseline age, BMI, sex, race, diabetes, hypertension, or dyslipidemia between responders and nonresponders at 6 months, 1 year, and 2 years. The number of responders and nonresponders for each time point are presented in Table 1.

**Table 1:** Responders and Nonresponders Among Participants in MOVE! Program (N = 375) at 2 Weeks, 4 Weeks, 6 Months, 1 Year, and 2 Years, Augusta, Georgia, 2008–2010^a^

Time	Available Data	Status					
**Time Initial Response Assessed**	**No. of Participants With Data on Weights Available**	**Initial Status**		**Status of Participants at 6 Months**		**Status of Initial Responders at 6 Months**	
		Achieved Weight Reduction (Responder)	Did Not Achieve Weight Reduction (Nonresponder)	Achieved 5% Weight Reduction (Responder)	Did Not Achieve 5% Weight Reduction (Nonresponder)	Achieved 5% Weight Reduction (Responder)	Did Not Achieve 5% Weight Reduction (Nonresponder)
2 weeks	83	35^b^	48	19	64	14	21
4 weeks	82	39^c^	43	19	63	16	23
**Time Initial Response Assessed**	**No. of Participants With Data on Weights Available**	**Initial Status**		**Status of Participants at 1 Year**		**Status of Initial Responders at 1 Year**	
		Achieved Weight Reduction (Responder)	Did Not Achieve Weight Reduction (Nonresponder)	Achieved 5% Weight Reduction (Responder)	Did Not Achieve 5% Weight Reduction (Nonresponder)	Achieved 5% Weight Reduction (Responder)	Did Not Achieve 5% Weight Reduction (Nonresponder)
2 weeks	107	51^b^	56	21	86	13	38
4 weeks	107	54^c^	53	19	88	16	38
**Time Initial Response Assessed**	**No. of Participants With Data on Weights Available**	**Initial Status**		**Status of Participants at 2 Years**		**Status of Initial Responders at 2 Years**	
		Achieved Weight Reduction (Responder)	Did Not Achieve Weight Reduction (Nonresponder)	Achieved 5% Weight Reduction (Responder)	Did Not Achieve 5% Weight Reduction (Nonresponder)	Achieved 5% Weight Reduction (Responder)	Did Not Achieve 5% Weight Reduction (Nonresponder)
2 weeks	102	51^b^	51	19	83	12	39
4 weeks	98	51^c^	47	20	78	12	39

^a^ Not all 375 participants had weights for each time point; for example, a participant could have had a weight at baseline, 2 weeks, and 6 months, but not at 1 year or 2 years.

^b^ Achieved ≥0.5% weight reduction at 2 weeks.

^c^ Achieved ≥1.0% weight reduction at 4 weeks.

Very early response predicted successful weight reduction (5%) at 6 months. Very early responders were more than 5 times as likely to achieve the 5% weight-reduction goal at 6 months as participants who were not very early responders (Table 2). However, very early response did not predict successful weight reduction at 1 year or at 2 years. Early response predicted successful weight reduction at 6 months and 1 year. Participants with an early response were more than 10 times as likely to meet the weight-reduction goal of at least 5% at 6 months as participants who did not respond early. In addition, early responders were almost 7 times as likely to meet the weight-reduction goal of at least 5% at 1 year as participants who did not respond early. Neither very early nor early response predicted successful weight reduction at 2 years.

**Table 2:** Likelihood of Successful Weight Reduction for Initial Responder Responders at 3 Points in Time, MOVE! Participants (N = 375), Augusta, Georgia, 2008–2010

Response	No. of Participants	Odds Ratio^a, b^ (95% Confidence Interval)	*P* Value
**Successful weight reduction of ≥5% at 6 months**			
≥0.5% at 2 weeks	83	5.46 (1.69–17.71)	.005
≥1.0% at 4 weeks	82	10.76 (2.64–43.80)	.001
**Successful weight reduction of ≥5% at 1 year**			
≥0.5% at 2 weeks	107	2.06 (0.75–5.69)	.16
≥1.0% at 4 weeks	107	6.96 (1.85–26.13)	.004
**Successful weight reduction of ≥5% at 2 years**			
≥0.5% at 2 weeks	102	1.68 (0.58–4.90)	.34
≥1.0% at 4 weeks	98	1.47 (0.52–4.11)	.47

^a^ Reference group for those who responded at 2 weeks or 4 weeks is those who did not respond at 2 weeks or 4 weeks, respectively.

^b^ Controlling for age, sex, race, and total number of sessions attended, which were nonsignificant contributors to the model.

Neither very early nor early response predicted the total number of sessions attended during the first 6 months (Table 3) or high-intensity participation (Table 4).

**Table 3:** Results of Linear Regression for Predicting Total Number of Sessions Attended During the First 6 Months of Program, by Initial Program Response, MOVE! Participants (n = 375), Augusta, Georgia, 2008–2010

Initial Response	No. of Participants	β (SE)	95% CI	*t*	*P* Value
**Participation in first 6 months of program based on initial response**					
≥0.5% at 2 weeks	126	0.86 (0.87)	−0.87 to 2.59	0.98	.33
≥1.0% at 4 weeks	123	0.48 (0.85)	−1.20 to 2.17	0.57	.57
**Participation in first 6 months of program for those with data on a 6-month weight**					
≥0.5% at 2 weeks	83	1.05 (1.19)	−1.33 to 3.43	0.88	.38
≥1.0% at 4 weeks	82	0.79 (1.14)	−1.48 to 3.05	0.69	.49

Abbreviations: β, slope; CI, confidence interval; SE, standard error.

**Table 4:** Results of Logistic Regression for Likelihood of Achieving High-Intensity Participation,^a^ by Initial Response, MOVE! Participants (n = 375), Augusta, Georgia, 2008–2010

Initial Response	No. of Participants	Odds Ratio (95% Confidence Interval)	*P* Value
**High-intensity participation in first 6 months of program**			
≥0.5% at 2 weeks	126	0.60 (0.17–2.17)	.44
≥1.0% at 4 weeks	123	1.04 (0.30–3.61)	.95
**High-intensity participation in first 6 months of program for those with data on a 6-month weight**			
≥0.5% at 2 weeks	83	0.66 (0.15–2.83)	.57
≥1.0% at 4 weeks	82	1.12 (0.30–4.20)	.87

^a^ Defined as attending 14 or more sessions within first 6 months.

## Discussion

We used clinical data from MOVE! participants who were most in need of weight reduction to examine the role of very early response and early response to the lifestyle intervention in later success with weight reduction and in program participation. A very early response predicted short-term (6 months) but not later (1 year or 2 years) achievement of weight-reduction goals. An early response predicted achievement of weight-reduction goals for 6-month and 1-year terms but not 2-year terms. Although the findings at 2 years could have resulted from successful weight reduction that led to healthier participants who needed health care less frequently and therefore were not represented in later samples, they are more likely due to the common phenomenon of weight regain after intervention completion (1). Participants and providers may consider a lifestyle intervention to be a bolus — to be taken intensively for a short period — rather than a long-term series of regular doses. We are not aware of any other reports that use the precise definitions of very early and early responder with which to compare our findings; however, our finding that an initial response predicted later success is consistent with the findings of other studies (3–7).

Previous reports on the MOVE! program indicated that participation and high-intensity participation were associated with successful weight reduction (8,9,12,13). However, our findings did not support the hypothesis that those who were experiencing weight reduction would continue to attend sessions while those who were not experiencing weight reduction would stay away. Neither very early nor early success with weight reduction predicted participation or high-intensity participation. We defined very early and early responders precisely; further study would be required to determine if participants are using some other definition of success to inform their decisions on participation. In addition, many other factors could influence participation. Program developers and health care providers may wish to capitalize on the health care system through which MOVE! is offered; opportunities for increasing participation may exist by engaging veterans during visits to Veterans Affairs facilities for other reasons.

Our findings have implications for policy makers and health care providers because they suggest that 2 weeks may be a good point at which to further tailor the MOVE! intervention. Because early responders were more likely to be successful at 6 months and at 1 year than participants who were not early responders, steps should be taken to assist participants to reach a 1.0% weight reduction at 1 month. Initial weight reduction should be assessed at 2 weeks. If participants are not on target at that point, a more thorough assessment should be conducted to determine how the health care provider could help. This help could require significantly more time from health care providers; a group of health care providers may need to be engaged at this point to address the participant’s needs. Rather than waiting 6 months to determine if the 5% weight reduction goal will be met, augmenting the lifestyle program may be appropriate at 2 weeks. In addition, further study is needed to determine if other differences (eg, genetics, personal health beliefs, cultural factors) exist between very early responders and early responders and nonresponders and how health care providers can tailor interventions to address those differences.

Our study had strengths and limitations. Among the strengths, using clinical data and following participants over time allowed us to examine the role of initial responses to the intervention on later participation and weight-reduction outcomes. Using percentage of weight reduction instead of pounds makes our findings more applicable to a wide range of baseline body sizes and is consistent with the clinical guidelines on setting the initial weight-reduction goal as a percentage (1,2). Although using clinical data from health care records of community-dwelling adults with decisional control lacks the requirement for measuring weights at specified times as one might see in a randomized clinical trial, it is a strength of the study because it is clinically realistic. Another strength is that because this was a retrospective study, participants had no opportunity to alter their behavior because they were being observed (8,9,14). Clinical data may not be complete or completely accurate, but they directly reflect the information available to clinicians and, therefore, findings from this study could easily be applied to practice (15). One important potential limitation is that the health care records may not have correctly classified the race/ethnicity of participants; race was not a significant predictor of successful weight reduction in this study. We did not examine other variables, such as dietary adherence, physical activity, or medications, which might also have influenced outcomes. In addition, we examined data on veterans most in need of a weight-reduction intervention. Examining data on participants less severely impaired by obesity, participants who are overweight but not obese, and participants who do not have comorbid conditions is an area for further research. Initial fluid loss may have confounded our findings on very early weight reduction; this factor should be explored with further study. Furthermore, our findings may not be generalizable to all comprehensive lifestyle programs or to populations other than veterans. Therefore, other comprehensive lifestyle programs should be examined to determine if these simple formulas of 0.5% weight reduction at 2 weeks and 1.0% weight reduction at 1 month have similar predictive value in other programs and among other groups.

We conclude that very early responders and early responders to MOVE! were more likely to achieve weight reduction at 6 months than participants who were not very early or early responders. Early responders were also more likely to continue to achieve weight reduction at 1 year. Neither very early response nor early response predicted the level of program participation. This information is particularly relevant for addressing policies aimed at reducing obesity and its comorbid conditions among veterans.
